# Anti-Diabetic Effects of Phenolic Extract from Rambutan Peels (*Nephelium lappaceum*) in High-Fat Diet and Streptozotocin-Induced Diabetic Mice

**DOI:** 10.3390/nu9080801

**Published:** 2017-07-26

**Authors:** Qingyu Ma, Yan Guo, Liping Sun, Yongliang Zhuang

**Affiliations:** Yunnan Institute of Food Safety, Kunming University of Science and Technology, No. 727 South Jingming Road, Kunming 650500, China; mqy0323@hotmail.com (Q.M.); guoyan8090@hotmail.com (Y.G.); lpsun@kmust.edu.cn (L.S.)

**Keywords:** rambutan peels, phenolics, anti-diabetic, hepatic glycogen, histological analysis, immunohistochemical

## Abstract

Recent studies have shown that rambutan peel phenolic (RPP) extract demonstrate high antioxidant and antiglycation activities in vitro and in vivo. This study further evaluated the anti-diabetic activity of RPP in a mouse model of Type II diabetes induced by streptozotocin combined with high-fat diet. Results showed that RPP increased the body weight and reduced the fasting blood glucose level of the diabetic mice. RPP significantly reduced the serum levels of total cholesterol, triglyceride, creatinine, and glycated serum protein in diabetic mice in a dose-dependent manner. Glycogen content in mice liver was recovered by RPP, which further increased the activity of superoxide dismutase and glutathione peroxidase and reduced lipid peroxidation in diabetic mice. Histological analysis showed that RPP effectively protected the tissue structure of the liver, kidney, and pancreas. In addition, RPP decreased the mesangial index and inhibited the expression of TGF-β in the kidney of diabetic mice.

## 1. Introduction

Diabetes mellitus (DM) is currently a major health problem worldwide. DM is the most common chronic disease characterized by elevated blood glucose levels [1]. Long-term exposure to high blood glucose levels results in production of reactive oxygen species. Oxidative stress is one of the main mechanisms of progression of diabetes and actively leads to cellular injury that precedes the onset of many diabetic complications [2]. Moreover, hyperglycemia is involved in the development of microvascular and macrovascular complications, which are the major causes of diabetes-related morbidity and mortality [3].

Researchers are developing a number of oral medicines to treat diabetes. However, these drugs demonstrate significant side effects, including weight gain and gastrointestinal distress. Therefore, finding new potential natural products that prevent DM is necessary [4]. Phenolic compounds are important secondary plant metabolites that determine the sensory and nutritional qualities of fruits, vegetables, and other plant products. Phenolics have received attention in recent years because of their antioxidant [5], antimicrobial [6], antimelanogenesis [7], hepatoprotective [8] and anti-inflammatory [9] effects. Studies suggest that a large number of phenolic extracts from plants, including mulberry leaf [10], *Pistachia lentiscus* L. leaves from Algeria [11], *Astilboides tabularis* [12], mate tea [13], Korean sorghum [14], *Pongamia pinnata* Pierreseeds [15], and *Pseuduvaria monticola* bark [16], demonstrate anti-diabetic properties.

We previously extracted crude rambutan phenolic peel (RPP) extract through microwave-assisted extraction, wherein the operating parameters were optimized. Our study showed that RPP possesses a potent free radical scavenging activity due to its phenolic contents [17]. Crude RPP extracts were purified using NKA-9 resin adsorption technology. After purification, phenolic content, along with some important phenolic compounds, such as geraniin (122.18 mg/g dry weight (dw)), procyanidin trimers (4.06 mg/g dw), procyanidin dimmers (11.60 mg/g dw), corilagin (7.56 mg/g dw), catechin (9.80 mg/g dw), and ellagic acid (9.31 mg/g dw), were enriched; additionally, the antioxidant and antiglycation activity in vitro were increased [18]. Purified RPP exerts high inhibitory oxidative stress on H_2_O_2_-induced HepG2 cells and anti-aging activity induced by D-galactose in vivo. Moreover, RPP effectively provides protection against D-gal-induced liver and kidney tissue damage in mice [19].

On the basis of the above results, we speculated that purified RPP can reduce blood glucose levels. However, no study has been conducted to scientifically prove the anti-diabetic activity of RPP. This study thus aims to demonstrate the anti-diabetic activity of RPP in animal models of diabetes induced by streptozotocin (STZ) combined with high-fat diet. 

## 2. Materials and Methods

### 2.1. Materials and Reagents

Purified RPP sample was prepared as previously described [18]. High-fat diets (45% fat) were purchased from Research Diets Inc. (New Brunswich, NJ, USA). Dimethylbiguanide hydrochloride (DMBG) was purchased from Sino-American Shanghai Squibb Pharmaceuticals Ltd. (Shanghai, China). STZ was purchased from Sigma-Aldrich (St. Louis, MO, USA). Commercial assay kits for creatinine (CRE), glycated serum protein (GSP), total cholesterol (TC), total triglycerides (TG), total protein (TP), hepatic glycogen (GC), superoxide dismutase (SOD), glutathione peroxidase (GSH-Px), and malonaldehyde (MDA) were obtained from Nanjing Jiancheng Biological Engineering Institute Co., Ltd. (Nanjing, China).

### 2.2. Experimental Animals

Male Institute of Cancer Research (ICR) mice (weighing 18–22 g) were provided by the Kunming Medical University Animal Research Center (Kunming, China). All mice were acclimatized for 7 days to the conditions of the animal room (ambient temperature of 20–25 °C and a 12 h-dark/12 h-light cycle) and provided with free access to standard pellet diets and water. All animal experiments were performed in strict accordance with the animal experimentation guidelines approved by the Animal Care and Use Committee of our Institute.

### 2.3. Experimental Design

#### 2.3.1. Modeling Method

After a week-long adaptive fitness program, all mice were fasted for 6 h, and eight mice were randomly selected as members of the normal group (NG). The NG mice were injected with 0.06 mL of saline, whereas the other mice were intraperitoneally injected with 0.06 mL of STZ at a dose of 50 mg/kg body weight (bw). This treatment was repeated at same time for 3 days. Afterward, the ordinary feed was changed into high-fat diet. The mice fed with high-fat diet were followed up for 14 days. The fasting blood glucose level of these mice was measured, and those showing a fasting blood glucose level higher than 11.1 mmol/L were considered hyperglycemic.

The hyperglycemic mice were randomly divided into five groups, each group consisting of eight animals; these groups were the positive control group (PG, 10 mg/kg bw DMBG taken orally daily), Model group (MG, saline taken orally daily), low-dose group (RPP-L, 50 mg/kg bw RPP taken orally daily), middle-dose group (RPP-M, 100 mg/kg bw RPP taken orally daily), and high-dose group (RPP-H, 200 mg/kg bw RPP taken orally daily). The mice were weighed to adjust the oral doses according to changes in their weight. The fasting blood glucose level of the mice was measured from the tail veins by using a glucometer (Bayer HealthCare LLC, Bayer, IN, USA), and the readings were recorded weekly for 5 weeks. 

#### 2.3.2. Biochemical Assays

Serum was collected from blood via centrifugation at 2000 r/min for 15 min at a temperature below 5 °C. Livers, kidneys, and pancreas were collected, and floating blood was washed out with ice-cold physiological saline.

Serum concentrations of CRE, GSP, TC, and TG were measured on an automatic biochemical analyzer (RaytoChemray 240, Shenzhen Rayto Co., Ltd., Shenzhen, China) by using commercial kits.

HG concentration was measured using a colorimetric method, whereas TP of liver was measured using the Bradford method. The levels of hepatic SOD, GSH-Px, and MDA were determined using commercial assay kits. All of the experimental procedures were performed strictly in accordance with the kit instructions.

#### 2.3.3. Histopathological and Immunohistochemical Analyses of Tissues

##### Histopathological Analysis of Tissues

Tissues samples were obtained from liver, kidney, and pancreas, fixed in 10% buffered formalin, dehydrated in alcohol, and embedded in paraffin. Paraffin sections (2–3 μm thick) were cut and stained with hematoxylin-eosin (HE). Histopathological changes in the liver, kidney, and pancreas were visualized using an Olympus DP70 Digital Camera System at 200× magnification.

The 2–3 μm-thick paraffin sections of the kidney were cut and stained with Periodic Acid-Schiff (PAS). The condition of the lesion in glomeruli and interstitial tubules was observed under an Olympus DP70 Digital Camera System at 200× magnification. 

##### Immunohistochemical Analysis of Tissues

Paraffin sections (2–3 μm thick) of kidney were blocked with 5% BSA solution after the process of microwave repair antigen. Subsequently, 100 μL of transforming growth factor-β1 (TGF-β1, 1:100) antibody was added into each slice at 4 °C overnight. After washing with tap water several times, 100 μL of sheep anti-mouse/rabbit IgG polymer was dropped on each slice, which was placed at room temperature for 15 min and then washed with phosphate buffer saline three times for 3 min each round. After being stained with diaminobenzidine, the slices were encapsulated in neutral resin film following conventional dewatering. The integral optical density of TGF-β1expression region was visualized with an Olympus DP70 Digital Camera System at 200× magnification and then measured using an image-pro Insight analysis software (Media cybernetics Inc., Rockville, MD, USA).

### 2.4. Statistical Analysis

Experimental data were presented as means ± SD (*n* = 8). Statistical significance of the difference between groups was detected using SPSS19.0 software (SPSS Inc., Chicago, IL, USA). *p* Values of <0.05 indicated statistical significance.

## 3. Results and Discussion

STZ produces oxygen free radicals in the body, resulting in selective pancreatic islet β-cell cytotoxicity and increased blood glucose level. Low-dose STZ induced metabolic characteristics of the human Type II diabetic mellitus (T2DM) [10]. Researchers have developed an animal model fed with low-dose STZ combined with high-fat diet to evaluate the effects of natural products on T2DM [20]. This model is potentially useful in studying the anti-diabetic properties of natural compounds of plant origin. In this study, mice with T2DM induced by high-fat diet combined with low-dose STZ injection were used to evaluate the anti-diabetic activity of RPP.

### 3.1. Body Weight

**Figure 1:**
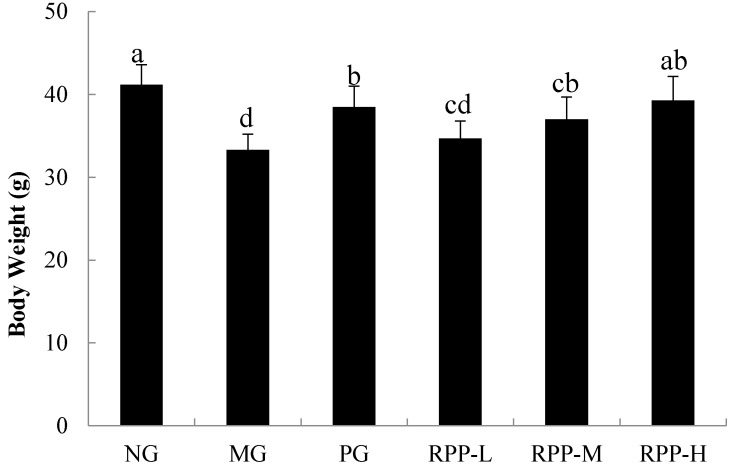
Effect of rambutan peel phenolic (RPP) on body weight of mice after 5 weeks. NG: Normal group; MG: streptozotocin (STZ), Model group; PG: STZ, DMBG 10 mg/kg; RPP-L: STZ, 50 mg/kg RPP; RPP-M: STZ, 100 mg/kg RPP; RPP-H: STZ, 200 mg/kg RPP. Different lower-case letters indicate significant differences (*p* < 0.05).

STZ-induced diabetes is characterized by a severe loss in body weight resulting from increased muscle destruction or degradation of structural proteins [21]. Compared with the body weight of NG mice, that of MG mice significantly decreased by 19.17% (Figure 1). When the diabetic mice were treated with RPP, their body weights improved. The body weight of RPP-M mice did not differ from that of PG mice (*p* > 0.05), the body weight of which was significantly higher than that of MG mice (*p* < 0.05). The body weight of RPP-H group did not differ with that of NG group (*p* < 0.05). These results indicated that RPP prevents body weight loss by controlling muscle wasting. Our results are consistent with those of a previous study [21].

### 3.2. Fasting Blood Glucose (FBG)

**Figure 2:**
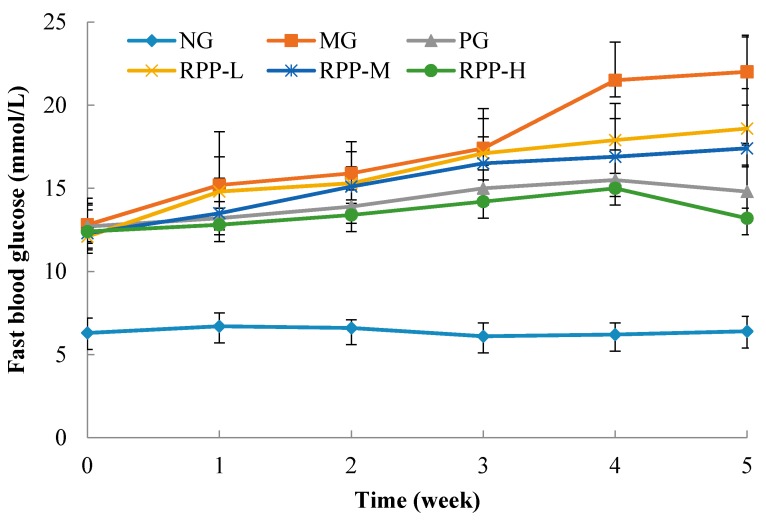
Effect of RPP on fast blood glucose level of mice. NG: Normal group; MG: STZ, Model group; PG: STZ, DMBG 10 mg/kg; RPP-L: STZ, 50 mg/kg RPP; RPP-M: STZ, 100 mg/kg RPP; RPP-H: STZ, 200 mg/kg RPP.

High FBG is a key indicator in diabetic mice. After STZ injection, FBG levels in MG mice were significantly higher than those in NG mice (*p* < 0.05) (Figure 2), indicating that a T2DM model was successfully built through STZ injection. These results suggest that RPP effectively inhibited the increase in FBG in T2DM, and the inhibitory activity increased with increased RPP concentration. RPP-H had stronger inhibitory ability than PG, indicating that RPP noticeably inhibited FBG in diabetic mice. Studies have shown that the products demonstrating antioxidant and antiglycation activities effectively inhibit the increase in FBG. Mehenni et al. studied that gallic acid, catechin and ellagic acid in *Pistacia lentiscus* were key compositions to regulate glucose in diabetic rat [22]. Thus, as we previously reported [17,18,19], RPP were rich in catechin and ellagic acid, so the FBG inhibitory activity of RPP was due to the phenolic compounds with high antioxidant and antiglycation activities.

### 3.3. Serum Biochemical Indicators

TC refers to the sum of all lipoprotein cholesterols in the blood and is an important indicator in analysis of blood lipid in clinical practice. The high level of cholesterol can lead to atherosclerosis, diabetes, and symptoms of kidney disease. TG is a type of fatty acid molecule consisting of long-chain fatty acids and glycerol [23]. Part of the sugar can be converted into TG by the liver in vivo. Moreover, high levels of TG can lead to high blood pressure, pancreatitis, in aggravated hepatitis, or other injuries. As shown in Table 1, TC and TG levels in STZ-induced MG mice considerably increased compared with the NG level (*p* < 0.05). The serum TC and TG levels in RPP groups significantly decreased in a dose-dependent manner. The TC and TG levels in RPP-H mice did not significantly differ from those in NG mice (*p* > 0.05).

CRE is an indicator of the toxin content of the blood and an important indicator of diabetic nephropathy [24]. As shown in Table 1, CRE content significantly increased (*p* < 0.05) in MG group and approximately twice that in NG mice. RPP obviously dose-dependently inhibited the increase in CRE content in diabetic mice. Relative to the CRE level in MG mice, that in RPP-M and RPP-H mice decreased by 27.13% and 46.93%, respectively.

GSP is produced during plasma protein and glucose enzyme saccharification. High levels of blood glucose lead to the production of high GSP levels in a positive correlated manner. GSP levels reflect the average blood glucose levels within 1–3 weeks before [25]. Table 1 shows that the GSP contents in MG mice were significantly higher than those in NG mice (*p* < 0.05), which proved high blood glucose increased the production of GSP in mice. RPP and DMBG significantly reduced the GSP level in STZ-induced diabetic mice (*p* < 0.05) in a dose-dependent manner. Compared with that in MG mice, the GSP levels in RPP-M, RPP-L, and RPP-H mice decreased by 6.68%, 24.36%, and 38.07%, respectively. The change of GSP content in the serum of diabetic mice after treatment with RPP was same to the change of FBG (Figure 2). The activity of RPP was due to the high antioxidant and antiglycation activities [17,18,19]. Our results were similar to the previous study [24].

### 3.4. Biochemical Indicators in the Liver

TP level decreased in the liver of diabetic mice (Table 2). Compared with that in NG mice, the protein level in MG mice decreased by 23.53% (*p* < 0.05). Protein level in the liver of diabetic mice improved after treatment with RPP. The protein levels in RPP-M, RPP-H, and PG mice did not significantly differ from that in NG mice (*p* > 0.05). This result was obtained due to the distinct metabolic alterations that led to a negative nitrogen balance, enhancing proteolysis and reducing protein synthesis [26].

GC is a type of macromolecular polysaccharide composed of glucose units and is mainly stored in the liver and muscles as standby energy. GC level in various tissues directly reflects insulin activity [27]. In this study, the GC content of the liver of the diabetic mice was markedly reduced. As shown in Table 2, the GC level in MG mice significantly decreased by 48.78% (*p* < 0.05) compared with that in NG mice. RPP dose-dependently increased the GC level in the liver of diabetic rats. The increase in GC levels under the three RPP doses did not significant differ (*p* > 0.05). RPP-M and RPP-H had no significant difference with PG mice (*p* > 0.05). 

Oxidative stress is one of the main mechanisms of progression of diabetes and actively leads to cellular injury that can precede the onset of many diabetic complications [28]. Long-term exposure to high glucose levels results in increased production of reactive oxygen species. Oxidative stress is generally considered a causative factor in the development of insulin resistance and diabetic complications .This study further evaluated the protective effect of RPP on antioxidant enzyme and liver lipid peroxide in mice.

SOD and GSH-Px enzymes are important in enzymatic defense system in vivo. SOD converts superoxide radicals into hydrogen peroxide, whereas GSH-Px converts hydrogen peroxide into other compounds in the presence of glutathione [29]. Excessive production of reactive oxygen in the serum of diabetic animals is possibly due to the observed marked reduction in SOD and GSH-Px concentrations. As shown in Table 2, SOD activity in MG mice decreased by 32.24% compared with that in NG mice. This result suggests that SOD activity is disrupted in diabetic mice. The SOD activity in PG mice did not significantly differ with that in NG mice (*p* > 0.05). RPP increased the SOD activity, which was significantly higher in RPP-H mice than in MG mice (*p* < 0.05), reaching 94.14% that of NG. The GSH-Px activity in MG mice decreased significantly compared with that in NG mice (*p* < 0.05). RPP recovered the GSH-Px activity. At the low dose of RPP, the GSH-Px activity in RPP-L did not significantly differ with that in NG (*p* > 0.05). Our previous studies showed RPP had high antioxidant activities and protected antioxidant enzymes of D-gal-induced aging mice in vivo [17,18,19]. Therefore, the high antioxidant activity of RPP was a mechanism to protect the antioxidant enzyme activity from diabetic animals.

STZ induces severe oxidative stress in diabetic animals and possibly induces the peroxidation of polyunsaturated fatty acids, leading to the formation of MDA, which is a by-product of lipid peroxidation [30]. In this study, MDA content of MG mice significantly increased by 47.46% compared with that in NG mice. RPP inhibited the increase in MDA content in a dose-dependent manner. The MDA levels in RPP-H did not significantly differ from that in NG (*p* > 0.05). The result was similar to previous study [22]. It indicated that the antioxidant activity of RPP inhibited lipid peroxide in the liver of diabetic animals. 

Regarding the literature available, catechin [31], geraniin [31], procyanidin [32] and ellagic acid [33] have been related to anti-diabetic activity, both in vivo and in vitro, and with the involvement of different action mechanisms. Our previous studies showed geraniin, procyanidin, catechin, and ellagic acid had high concentrations in RPP [19]. Therefore, anti-diabetic activity of RPP was due to its phenolic compounds.

## 4. Histopathology

### 4.1. HE staining of Liver

**Figure 3:**
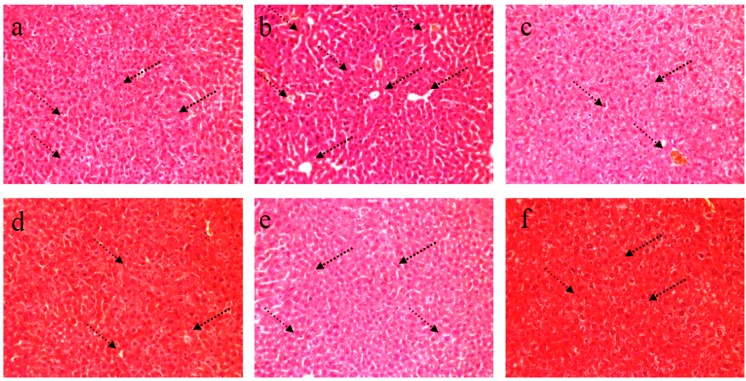
Effect of RPP on liver histology (H&E stain, 200×). (**a**) NG (Normal group); (**b**) MG (STZ, Model group); (**c**) PG (STZ, DMBG 10 mg/kg); (**d**) RPP-L(STZ, 50 mg/kg RPP); (**e**) RPP-M (STZ, 100 mg/kg RPP); (**f**) RPP-H (STZ, 200 mg/kg RPP).

Figure 3a shows the normal hepatic architecture of the mice. The hepatocytes showed distinct cell borders, and the central vein showed a round nucleus, which is surrounded by abundant cytoplasm. In MG mice (Figure 3b), the STZ-induced diabetic mice showed mussy hepatic cords. The hepatic nucleus presented serious pathological damage. The intercellular space increased, and deterioration in terms of size and shape were serious. Other damages, including focal necrosis, congestion in central vein, and infiltration of lymphocytes, were also observed. The PG group (Figure 3c) showed a normal hepatic architecture, and changes in size and shape of the hepatic cells were not evident. RPP treatment alleviated the pathological damage in the experimental groups compared with that in MG mice. In RPP groups (Figure 3d–f), RPP demonstrated a dose-dependent protective effect on the STZ-induced diabetic mice. RPP-H apparently effectively alleviated the symptoms of focal necrosis, congestion in central vein, and infiltration of lymphocytes. 

### 4.2. HE Staining of Kidney

**Figure 4:**
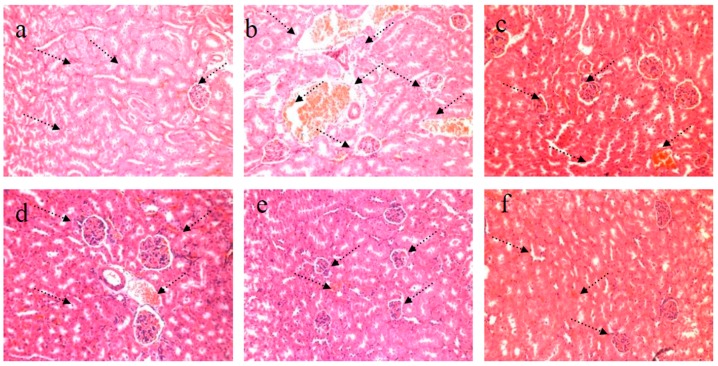
Effect of RPP on kidney histology (H&E stain, 200×). (**a**) NG (Normal group); (**b**) MG (STZ, Model group); (**c**) PG (STZ, DMBG 10 mg/kg); (**d**) RPP-L(STZ, 50 mg/kg RPP); (**e**) RPP-M (STZ, 100 mg/kg RPP); (**f**) RPP-H (STZ, 200 mg/kg RPP).

As shown in Figure 4a, the pathological tissue section of kidney revealed the distinct structure of both cortex and medulla in NG mice. The regular shape of glomeruli, renal tubule, and collecting duct was distinguished easily. The medulla displayed a distinctly ordered and packed arrangement. Moreover, symptoms of hemangiectasis, congestion, and inflammatory cell infiltration were not observed in the interstitial part. As presented in Figure 4b, the renal cortex and medulla of the STZ-induced diabetic MG mice showed varying degrees of atrophy. The cortex and medulla showed an architecture characterized by irregular distribution. The number of glomeruli declined obviously, and very serious glomerular sclerosis and expansion of kidney tubules were observed. The symptoms of inflammatory cell infiltration and congestion in central vein indicated a serious condition. Massive inflammatory cells infiltrated the glomeruli. The STZ-induced diabetic PG mice were treated with DMBG (Figure 4c). Glomerular degradation was alleviated to some degree. However, inflammatory cells still infiltrated the glomerular cells. Moreover, the medulla area is closely packed. In RPP experimental groups, diabetic nephropathy was relieved in different degrees after treatment with different RPP concentrations, especially the symptoms of congestion in central vein and inflammatory cell infiltration. This protective effect was dose dependent. RPP-H demonstrated the best effect on STZ-induced diabetic mice and restored the condition of the mice to nearly normal condition. This result indicated that RPP provided protection against STZ-induced kidney damage in diabetic mice.

### 4.3. HE Staining of Pancreas

**Figure 5:**
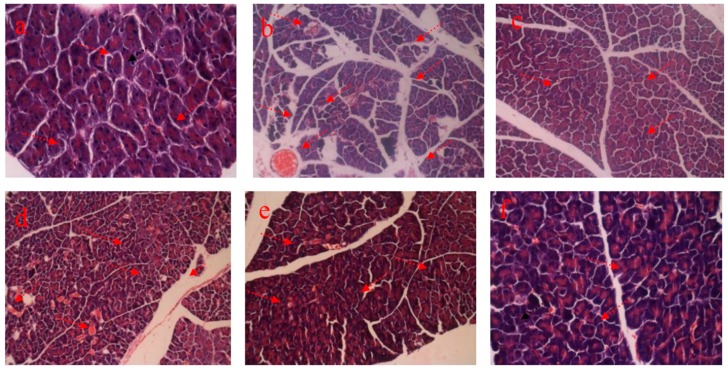
Effect of RPP on pancreas histology (H&E stain, 200×). (**a**) NG (Normal group); (**b**) MG (STZ, Model group); (**c**) PG (STZ, DMBG 10 mg/kg); (**d**) RPP-L(STZ, 50 mg/kg RPP); (**e**) RPP-M (STZ, 100 mg/kg RPP); (**f**) RPP-H (STZ, 200 mg/kg RPP).

As shown in Figure 5a, the pancreatic cells in NG mice showed a compact and ordered arrangement, as well as displayed a regular shape. Moreover, intercellular spaces were distributed uniformly, and congestion in central vein was not obvious. In MG mice (Figure 4b), serious pathological damages, such as focal necrosis, congestion in central vein, and infiltration of lymphocytes, were observed. In addition, the pancreatic cells showed a seriously altered shape, and they showed very irregular distribution. The pathology of pancreatic tissue generally demonstrates the varying degrees of damages in STZ-induced diabetic mice. In PG mice, the shape and size of the pancreatic cells was relatively homogeneous and are orderly packed. Moreover, the conditions of focal necrosis and infiltration of lymphocytes improved obviously (Figure 5c). The alleviation of pancreatic pathological damages varied in a dose-dependent manner in RPP-treated groups. As shown in Figure 4d–f, the protective effect of RPP-H was significantly better than that of RPP-L and RPP-M. In RPP-H mice, histological damage was notably mitigated and the conditions were restored to nearly the normal state. 

### 4.4. PAS Staining of Kidney

**Figure 6:**
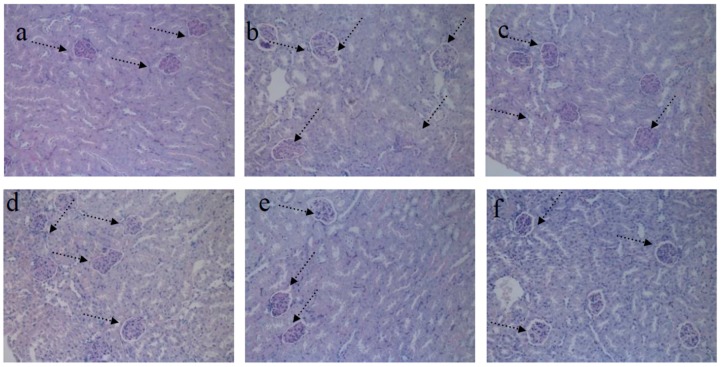
Effect of RPP on the mesangial index of kidney (PAS stain, 200×) (**a**) NG (Normal group); (**b**) MG (STZ, Model group); (**c**) PG (STZ, DMBG 10 mg/kg); (**d**) RPP-L(STZ, 50 mg/kg RPP); (**e**) RPP-M (STZ, 100 mg/kg RPP); (**f**) RPP-H (STZ, 200 mg/kg RPP).

After PAS staining, the cross-sectional area of glomeruli and glomeruli mesangial were analyzed by the Image-pro Insight analysis software (Media cybernetics Inc., Rockville, MD, USA). Mesangial matrix index, expressed as the ratio of the mesangial area to glomerular area, is an important indicator in evaluating kidney damage in diabetic mice. The kidney of STZ-induced diabetic mice showed glomerular enlargement and increased mesangial proliferation and mesangial index. As shown in Figure 6, the index in MG mice is obviously higher than that in NG mice. RPP treatment in each group mitigated histological damage and restored the mesangial index, thereby alleviating the symptoms of diabetes in mice. This protective effect was dose dependent. These results suggested that the effect of RPP on mesangial matrixis one of the key mechanisms in protecting the exterior of the kidney of STZ-induced diabetic mice.

### 4.5. TGF-β1 Staining of Kidney

**Figure 7:**
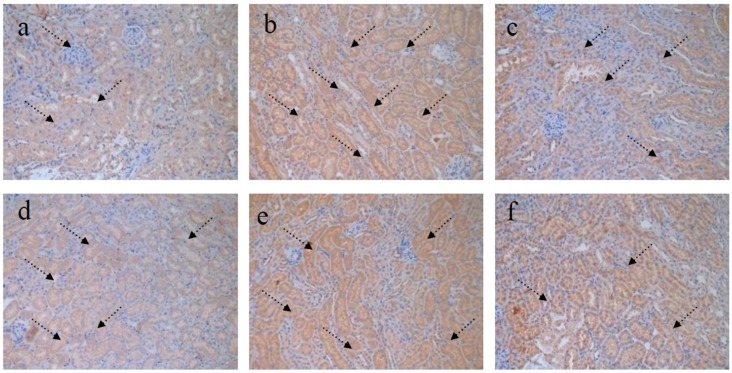
Effect of RPP on the TGF-β1of kidney (TGF-β1 stain, 200×). (**a**) NG (Normal group); (**b**) MG (STZ, Model group); (**c**) PG (STZ, DMBG 10 mg/kg); (**d**) RPP-L(STZ, 50 mg/kg RPP); (**e**) RPP-M (STZ, 100 mg/kg RPP); (**f**) RPP-H (STZ, 200 mg/kg RPP).

TGF-β1 is an immunosuppressant that inhibits the growth of T cells and B cells.TGF-β1 plays an important role in regulating cell growth, differentiation, and immune function [34]. In this study, immunohistochemical staining of mice kidney with TGF-β1were evaluated by Image-pro insight analysis software (Media cybernetics Inc., Rockville, MD, USA). The results in Figure 7 show that TGF-β1 expression in MG mice increased markedly compared with that in NG mice. The RPP-treated groups showed reduced TGF-β1 expression. As RPP dose increases, the effect of RPP on TGF-β1 expression of mice kidney also increases. TGF-β1 expression in RPP-H did not significantly differ with that in NG. TGF-β1 expression is possibly another protective mechanism of RPP in STZ-induced diabetic mice.

## 5. Conclusions

In this study, T2DMmice model was successfully developed through feeding with low-dose STZ combined with high-fat diet. The effect of RPP on biochemical indicators of serum and liver in mice were determined. Furthermore, histopathology of liver, kidney, and pancreas and immunohistochemistry of kidney were evaluated. The results indicated that RPP effectively reduced the damage in STZ-induced diabetic mice. This study introduced methods and provided data as basis for the potential applications of RPP as pharmaceutical and food ingredient.

## Figures and Tables

**Table 1:** Effect of RPP on biochemical indicators of mice serum.

Group	TC (mmol/L)	TG (mmol/L)	CRE (μmol/L)	GSP (mmol/L)
NG	5.90 ± 0.31d	1.43 ± 0.11c	10.62 ± 1.53d	7.14 ± 0.43d
MG	8.57 ± 0.43a	2.27 ± 0.13a	22.78 ± 1.96a	14.08 ± 1.12a
PG	6.28 ± 0.26c	1.53 ± 0.09c	13.93 ± 0.98c	7.93 ± 0.62d
RPP-L	7.12 ± 0.11bc	1.74 ± 0.11b	20.50 ± 2.04a	13.14 ± 0.86b
RPP-M	6.37 ± 0.35c	1.54 ± 0.10c	16.60 ± 1.14b	10.65 ± 0.91bc
RPP-H	6.01 ± 0.26cd	1.46 ± 0.12c	12.09 ± 0.85c	8.72 ± 0.78c

NG: Normal group; MG: STZ, Model group; PG: STZ, DMBG 10 mg/kg; RPP-L: STZ, 50 mg/kg RPP; RPP-M: STZ, 100 mg/kg RPP; RPP-H: STZ, 200 mg/kg RPP; TC: total cholesterol, TG: total triglycerides; CRE: creatinine; GSP: glycated serum protein. Different lower-case letters indicate significant differences (*p* < 0.05).

**Table 2:** Effect of RPP on biochemical indicators in mice liver.

Group	TP (g/g Liver)	GC (mg/g Liver)	SOD (U/mg)	GSH-Px (U/mg)	MDA (nmol/mg)
NG	0.17 ± 0.01a	32.37 ± 6.15a	316.19 ± 24.06a	105.11 ± 9.02a	1.18 ± 0.18c
MG	0.13 ± 0.02b	16.58 ± 4.76c	214.26 ± 17.10d	67.13 ± 7.00b	1.74 ± 0.37a
PG	0.16 ± 0.01a	27.70 ± 3.92b	305.60 ± 31.74a	101.58 ± 12.10a	1.36 ± 0.07b
RPP-L	0.13 ± 0.01b	19.62 ± 4.11bc	256.82 ± 31.63c	85.18 ± 13.64ab	1.70 ± 0.16a
RPP-M	0.16 ± 0.01a	22.22 ± 5.26b	263.69 ± 25.11bc	97.76 ± 6.30a	1.45 ± 0.24b
RPP-H	0.16 ± 0.01a	25.05 ± 4.71b	297.65 ± 23.29b	101.83 ± 10.60a	1.17 ± 0.16c

NG: Normal group; MG: STZ, Model group; PG: STZ, DMBG 10 mg/kg; RPP-L: STZ, 50 mg/kg RPP; RPP-M: STZ, 100 mg/kg RPP; RPP-H: STZ, 200 mg/kg RPP; TP: total protein; GC: hepatic glycogen; SOD: superoxide dismutase; GSH-Px: glutathione peroxidase; MDA: malonaldehyde. Different lower-case letters indicate significant differences (*p* < 0.05).
